# Intraosseous meningioma mimicking osteosarcoma

**DOI:** 10.4322/acr.2021.332

**Published:** 2021-10-29

**Authors:** Ruben Delgado, Hisham F. Bahmad, Vinay Bhatia, Allen B. Kantrowitz, Cristina Vincentelli

**Affiliations:** 1 Mount Sinai Medical Center, The Arkadi M. Rywlin M.D. Department of Pathology and Laboratory Medicine, Miami Beach, FL, USA; 2 Mount Sinai Medical Center, Department of Diagnostic Radiology, Miami Beach, FL, USA; 3 Mount Sinai Medical Center, Division of Neurosurgery, Miami Beach, FL, USA; 4 Florida International University, Herbert Wertheim College of Medicine, Miami, FL, USA

**Keywords:** Case Reports, Meningioma, Osteosarcoma, Skull

## Abstract

**Background:**

Predominantly intraosseous meningiomas are rare entities that include true primary intraosseous meningiomas (PIM), as well as meningiomas that may show extensive bone involvement, such as *en plaque* meningiomas. Different hypotheses have been proposed to decipher the origin of PIMs, such as ectopic arachnoid cap cell entrapment during birth or after trauma. Surgical resection is the treatment of choice of such lesions.

**Case presentation:**

We present a case of a 65-year-old man with an enlarging mass in the parieto-occipital region that grew slowly and progressively over 13 years, following head trauma during a motor vehicle accident. One year prior to presentation, he started experiencing daily holocranial headaches and blurry vision. CT and MRI studies revealed a permeative midline calvarial lesion measuring 14 cm in greatest dimension with extensive periosteal reaction, extension into the subcutaneous soft tissues, subjacent dural thickening and intracranial extension with invasion of the superior sagittal sinus. The favored pre-operative clinical diagnosis was osteosarcoma. The abnormal calvarium was excised and histopathological examination confirmed the diagnosis of a predominantly intraosseous calvarial meningioma, WHO grade I.

**Conclusions:**

The present case highlights the importance of histopathologic diagnosis in guiding therapeutic decisions and reiterates the necessity of considering PIM or meningiomas with extensive intraosseous component in the differential diagnosis of calvarial masses, even when imaging suggests a neoplasm with aggressive behavior, such as osteosarcoma.

## INTRODUCTION

Meningiomas are slow growing, generally benign tumors of the meninges arising primarily from meningothelial arachnoid cells.^1^ They are among the most common intracranial adult tumors with an estimated annual incidence of 78.6 cases per 1 million people.^1^ The clinical presentation of meningiomas is highly dependent on the location of the tumor. Intracranial meningiomas most commonly occur over the cerebral convexity and parasagittal region.^2^ Although most meningiomas arise from the arachnoid cap cells of the arachnoid layer,^3^ extradural meningiomas can occur but are relatively rare, accounting for less than 2% of all meningiomas.^4^^,^^5^ Around 80% of meningiomas can be cured by surgical resection; and the most relevant prognostic factor remains the extent of resection (assessed by the Simpson grading scale).^1^

Extracranial or extradural meningiomas including primary intraosseous meningiomas (PIM) of the skull have been also reported,^6^^,^^7^ with an estimated rate of 2.4% according to a study including 1088 meningioma cases.^7^ The minimal presence or complete absence of dural involvement in PIM makes establishing a correct diagnosis challenging, where differential diagnosis might include fibrous dysplasia, Paget’s disease, and osteosclerotic lesions (e.g., osteoma or osteosarcoma). Unusual presentations also include carpet-like dural-based meningiomas (*en plaque*), which may invade bone and result in extensive hyperostosis.^8^^,^^9^ Meningioma *en plaque* (MEP) represents a morphological variant characterized by a sheet-like lesion that infiltrates the dura and sometimes invades the bone.^10^ The term “*en plaque*” was first used by Cushing and Eisenhardt to differentiate MEP from the most common form of meningioma (“en masse”).^11^ MEP accounts for approximately 2-4% of all meningiomas,^8^ and typically arises in the spheno-orbital region, with convexity MEP being comparatively rarer.^12^ The morphologic features of MEP are usually those of a World Health Organization (WHO) grade I meningioma.^8^ Bone and soft tissue invasion are features that may be present and, in the absence of other criteria, do not warrant a higher WHO grade.^13^ Some intracranial meningiomas may as well extend to skull leading to cranial hyperostosis, but the meningioma per se will still be referred to as intracranial meningioma rather than extradural meningioma.^8^ Hyperostosis of the adjacent skull is a well-known finding in cases of meningioma, and is observed in 4.5% of all types, but is more frequently present in MEP with an occurrence of 13% to 49%.^9^

**Figure 1:**
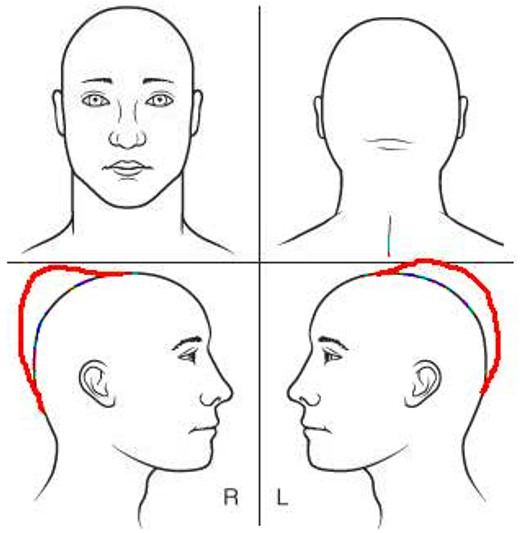
Schematic showing the location of the cranial mass in the patient’s parieto-occipital region.

**Figure 2:**
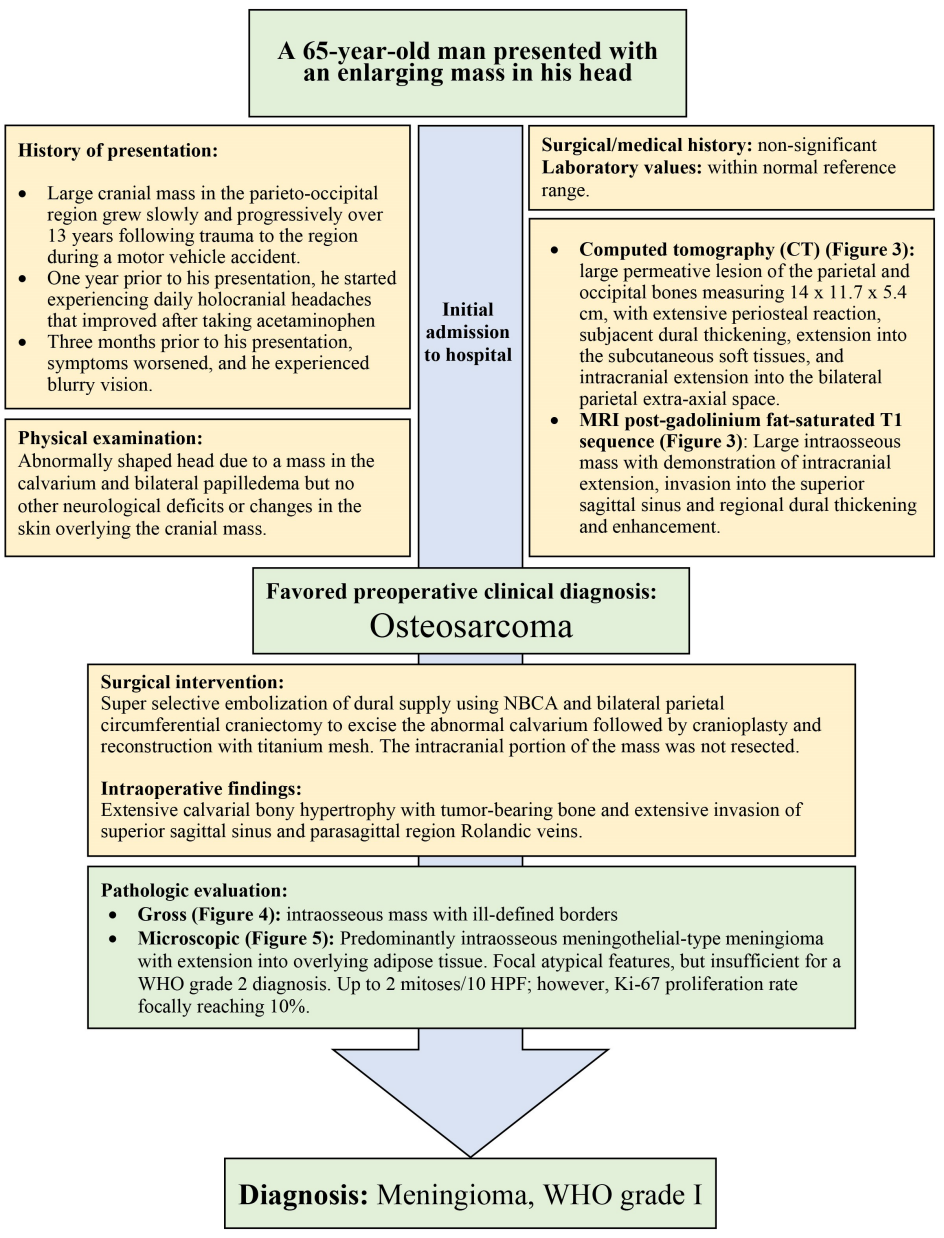
Summary of case timeline.

We present a case of a 65-year-old man with a large intraosseous calvarial mass in the parieto-occipital region that grew progressively over 13 years following head trauma (Figure 1). Imaging studies were performed showing an extensively destructive “bone tumor” and a preoperative clinical diagnosis of osteosarcoma was favored. The patient underwent circumferential craniectomy to excise the abnormal calvarium. Histopathologic evaluation revealed a predominantly intraosseous WHO grade I meningioma with extension into subcutaneous tissue and involving the superior sagittal sinus (Figure 2).

## CASE REPORT

A 65-year-old man presented with a chief complaint of daily, persistent holocranial headaches and blurry vision. Upon review of systems, the patient manifested having a cranial mass located in the parieto-occipital region for the past 13 years (Figure 1), which he believed had developed after head trauma he suffered in a motor vehicle accident.

The mass grew slowly and progressively over the years. It had never caused him any significant discomfort until approximately one year prior to presentation, when he started experiencing daily headaches which worsened with time and developed blurry vision. The patient's medical history also included essential hypertension, and he had no pertinent surgical, family or social history. On physical examination, an abnormally shaped head was noted due to a mass in the calvarium, as well as bilateral papilledema. There were no neurological deficits or changes in the skin overlying the cranial mass. The mass was not tender to palpation. Laboratory tests were within the normal reference range.

**Figure 3:**
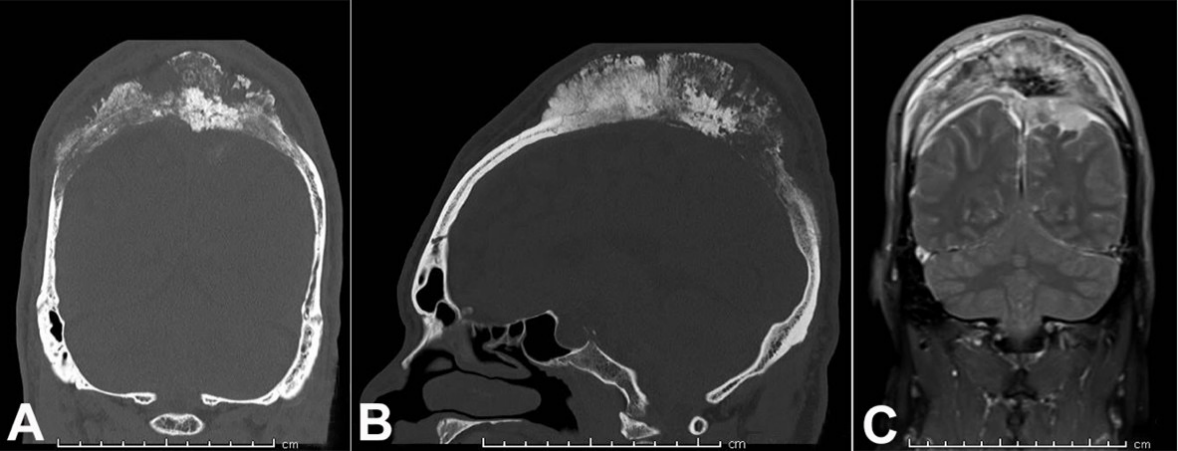
**A** and **B** (coronal and sagittal views respectively) – Bone computed tomography (CT). Large midline permeative calvarial lesion with areas of thickening/erosion of the inner table and extensive periosteal reaction; **C –** Coronal post-gadolinium fat-saturated T1 sequence. Large intraosseous mass with demonstration of intracranial extension, invasion into the superior sagittal sinus, and regional dural thickening and enhancement.

Computed tomography (CT) of the head showed a large midline permeative calvarial lesion measuring 14 × 11.7 × 5.4 cm, with extensive periosteal reaction, subjacent dural thickening, extension into the subcutaneous soft tissues and intracranial extension into the bilateral parietal extra-axial space (Figure 3).

CT-Angiography revealed invasion and occlusion of a 9.5 cm segment of the superior sagittal sinus. There was no evidence of infarction. Digital subtraction angiography reported a highly hyper-neovascular destructive bone tumor with exuberant blood supply from the bilateral occipital, superficial temporal, and middle meningeal arteries and pial supply from the posterior internal frontal branches of the left anterior cerebral artery.

Magnetic resonance imaging (MRI) post-gadolinium fat-saturated T1 sequence showed a large intraosseous mass with demonstration of intracranial extension, invasion into the superior sagittal sinus and regional dural thickening and enhancement (Figure 3). The favored pre-operative clinical diagnosis was osteosarcoma.

The patient underwent super selective embolization of dural supply using n-butyl 2-cyanoacrylate (NBCA) and bilateral parietal circumferential craniectomy to excise the abnormal calvarium, followed by cranioplasty and reconstruction with titanium mesh.

**Figure 4:**
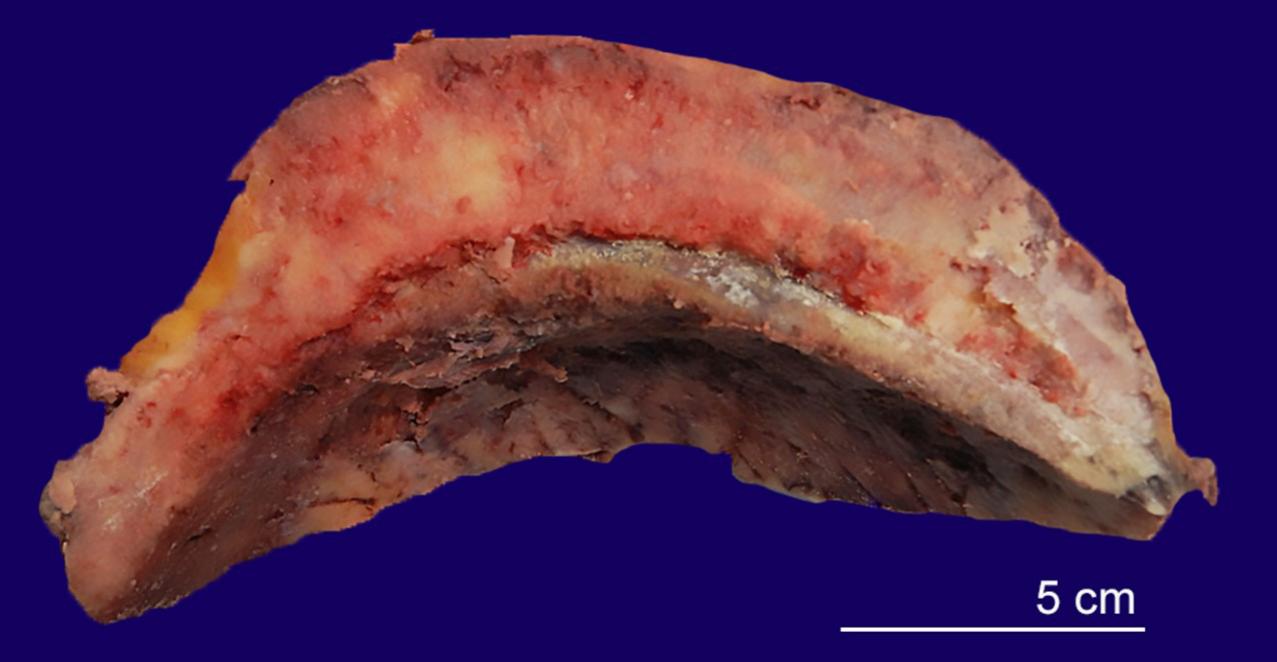
Gross examination of the resected bone. The image shows a fragment of calvarium measuring 22.5 cm in its longest axis, 7 cm in the coronal plane, and up to 1.5 cm in thickness. The bone is expanded by a mass.

An ovoid fragment of flat bone was received for pathologic examination. The outer surface of the specimen was nodular and had attached soft tissue, while the inner surface appeared flattened, irregular, and hemorrhagic. Sectioning revealed an ill-defined intraosseous mass expanding the bone. No areas of necrosis were grossly identified (Figure 4).

**Figure 5:**
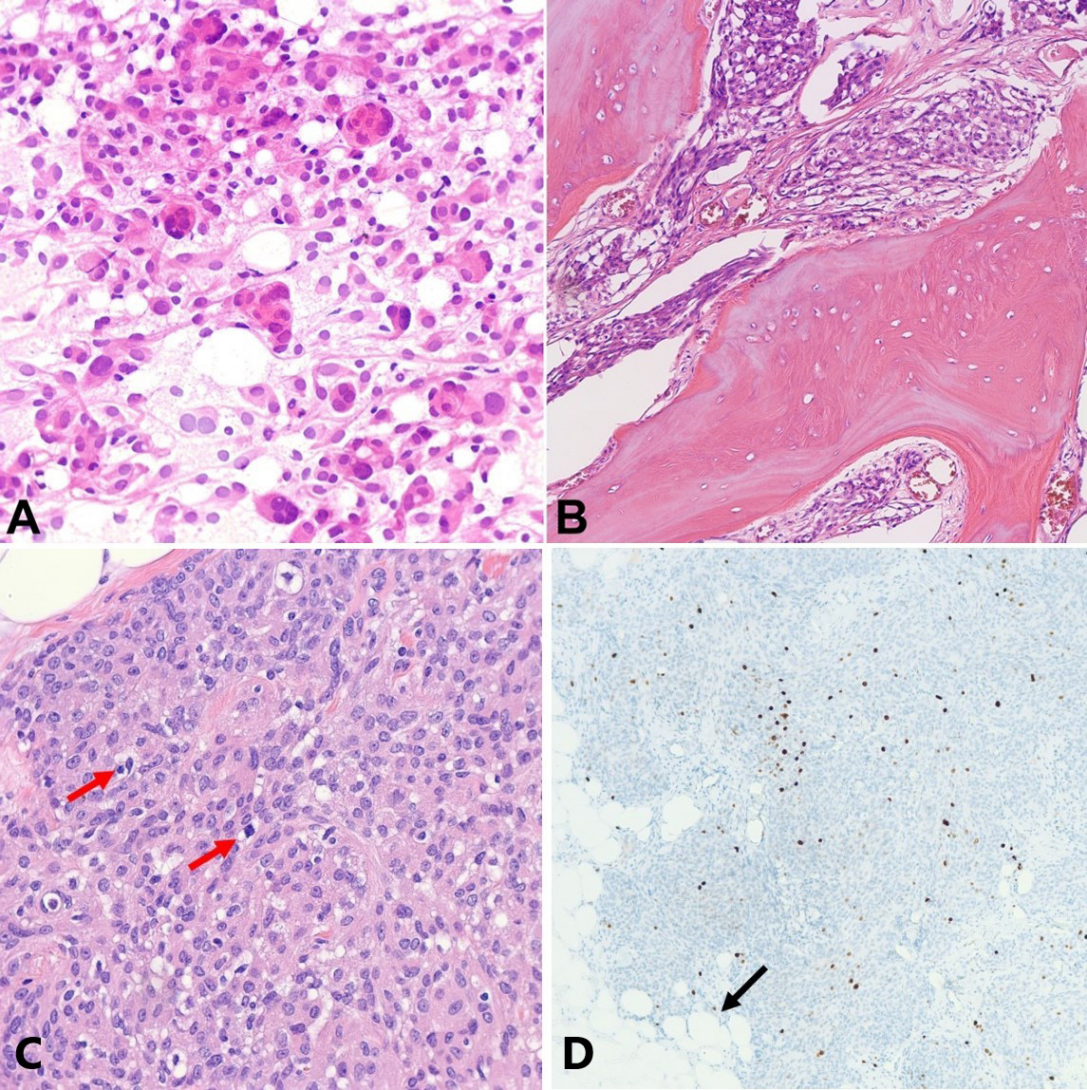
Photomicrographs of the tumor. **A –** Smear preparation showing cells with meningothelial differentiation forming whorls (H&E, 400x); **B –** predominantly intraosseous tumor with associated hyperostosis (H&E 200x); **C –** few scattered mitotic figures (red arrows) (H&E 400x); **D –** focally elevated Ki-67 proliferation rate (100x); and invasion into adipose tissue (arrow).

Microscopic examination of H&E-stained sections revealed a predominantly intraosseous meningioma with overall features of meningothelial-type meningioma, WHO grade I, composed of cells with eosinophilic cytoplasm and regular, ovoid nuclei with fine chromatin and small nucleoli arranged in syncytia and whorls, with extension into soft tissue (Figure 5).

Brain invasion was absent. Few mitotic figures were identified reaching up to 2 mitoses per 10 HPF. Atypical features were focally present, including a minute focus of incipient necrosis and an area of small cell change, and some cells showed mildly enlarged nucleoli. However, the findings were considered insufficient for a diagnosis of atypical meningioma, WHO grade II. The Ki-67 proliferation index was estimated to be less than 5% in average; however, it was noted that it was higher than expected for a usual WHO grade I meningioma reaching up to 10% in the most proliferative areas. The gross and microscopic histopathologic features were those of an *en plaque* meningioma with extensive bone invasion, or possibly a primary intradiploic (intraosseous) meningioma.

## DISCUSSION

Extracranial meningiomas are rare entities that arise outside the dural compartment. different nomenclatures have been used to describe them, including ectopic, extradural, calvarial, cutaneous, extraneuraxial or intraosseous meningiomas.^5^^,^^14^ In 2000, Lang et al.^15^ proposed the term ‘primary extradural meningioma’ (PEM) to refer to those lesions. PEMs can be subdivided into purely extradural (Type I), purely calvarial (Type II) or calvarial with extradural extension (Type III).^5^

In our case, the patient presented with a large destructive osseous mass invading the overlying soft tissue and intracranial structures, with prominent periosteal reaction and exuberant vasculature concerning for a malignant process. Although the pre-operative diagnosis was concerning for osteosarcoma, histopathological examination confirmed the diagnosis of a predominantly intraosseous calvarial meningioma, WHO grade I. The gross and microscopic features, taken together with the imaging findings, were those of an *en plaque* meningioma with extensive bone invasion, or possibly a primary intraosseous meningioma.

There have been different hypotheses proposed in the origin of primary extradural and calvarial meningiomas.^5^ Some authors have proposed that these arise from ectopic arachnoid cap cells trapped within the cranial sutures during molding of the head at birth,^16^ or due to misplacement after trauma where they get trapped in fracture lines.^17^ This hypothesis could apply to our case, given that the patient believed that the mass developed after head trauma he suffered during a motor vehicle accident. On the other hand, the origin of primary cutaneous meningiomas is thought to be either the result of arachnoid cell rests located in the skin due to defective closure of the neural tube,^18^ or multipotent mesenchymal cells as a reaction to an unidentified stimulus.^19^

As mentioned previously, it is also possible that the findings represent an *en plaque* meningioma with extensive invasion into the overlying calvarium. What is noteworthy about this case is that meningiomas with extensive intraosseous component, either due to bone invasion or primary intraosseous origin, may mimic bone malignancies such as osteosarcoma on imaging, even when these are benign neoplasms (WHO grade I meningiomas).

## CONCLUSION

Our case highlights the importance of histopathologic diagnosis in guiding therapeutic decisions and reiterates the necessity of considering this entity in the differential diagnosis of calvarial masses, even when imaging is suggestive of aggressive behavior. Therapeutic options for intraosseous calvarial meningiomas include surgical resection as the treatment of choice, with radiotherapy in case there is evidence of rapid disease progression.^5^
